# Trends in Prevalence and Incidence of Epilepsy and Drug-Resistant Epilepsy in Children: A Nationwide Population-Based Study in Korea

**DOI:** 10.3390/neurolint16040066

**Published:** 2024-08-21

**Authors:** Jooyoung Lee, Arum Choi, Sukil Kim, Il Han Yoo

**Affiliations:** 1 Department of Pediatrics, Eunpyeong St. Mary’s Hospital, College of Medicine, The Catholic University of Korea, Seoul 03312, Republic of Korea; jy37jy@catholic.ac.kr; 2 Department of Preventive Medicine and Public Health, College of Medicine, The Catholic University of Korea, Seoul 06591, Republic of Korea; dyemelody@gmail.com (A.C.); sikimmd@catholic.ac.kr (S.K.); 3 Department of Pediatrics, St. Vincent’s Hospital, College of Medicine, The Catholic University of Korea, 93 Joongbu-daero, Paldal-gu, Suwon-si 16247, Republic of Korea

**Keywords:** epidemiology, pediatric epilepsy, intractable epilepsy, administrative data, anti-seizure medication

## Abstract

Population-based data on drug-resistant epilepsy (DRE) are lacking. This retrospective study aimed to determine the prevalence and incidence of pediatric epilepsy and DRE in South Korea using health insurance claims data from the Health Insurance Review and Assessment Service (2013–2022). Epilepsy and DRE prevalence and incidence in children <18 years old were estimated over time and by age and sex. Results showed that the age-standardized prevalence and incidence rates of epilepsy increased. The age-standardized prevalence rate of DRE increased, while the age-standardized incidence rate remained unchanged. The standardized prevalence rate of DRE was 0.26 per 1000 persons, and the average standardized incidence rate of DRE was 0.06 per 1000 persons. The prevalence rate of DRE gradually increased with age, with age 0 demonstrating the highest incidence rate. The prevalence of generalized DRE was the highest across all ages, and incidence was the highest at 0 years. Conversely, the incidence of focal DRE did not change with age. Our study revealed a stable incidence rate of DRE in Korea, despite increased prevalence. DRE incidence was the highest in the first year of life, with the generalized type being the most prevalent.

## 1. Introduction

Epilepsy affects approximately 1% of the population [1], and approximately two-thirds of these patients are usually free from seizures using anti-seizure medications (ASMs) [2]. However, some patients experience persistent seizures despite treatment with ASMs. These patients are diagnosed with drug-resistant epilepsy (DRE), defined by the International League Against Epilepsy as a “failure to achieve sustained seizure freedom after adequate trials of two tolerated and appropriate medications [3]”. DRE is often diagnosed in pediatric and older patients [4], with a recent meta-analysis of 103 studies reporting a cumulative DRE incidence of 25.0% in pediatric populations and 14.6% in adult/mixed-age populations [5].

DRE is particularly common in children with developmental and epileptic encephalopathies such as Lennox–Gastaut syndrome and Dravet syndrome [6,7]. Predictors of DRE include early seizure onset, multiple seizure types, frequent seizures, status epilepticus, brain abnormalities, global developmental delay, and intellectual disability [4,8,9,10]. DRE negatively affects individuals and society. Patients with DRE are more susceptible to physical or psychiatric complications and the condition is associated with a high incidence of sudden, unexpected death. Additionally, a diagnosis of DRE contributes to increased healthcare costs due to the frequent utilization of resources [11,12,13].

Understanding the epidemiological characteristics of intractable epilepsy in children is essential for improving prevention, diagnosis, treatment, and management strategies. However, there is a significant lack of nationwide studies on DRE epidemiology, especially in Asian populations where data are limited and inconsistent. Most existing research has focused on specific populations or healthcare settings, hindering the generalizability of findings [14,15]. To investigate the prevalence and incidence of DRE in children, this study leverages nationwide health insurance claims data from South Korea, a population that has not been extensively studied in the existing literature. By analyzing trends over a decade, this research provides novel insights into the epidemiology of DRE in Asian populations. Moreover, this study aims to determine the prevalence and incidence rates, annual percentage change (APC), and average annual percentage change (AAPC) of DRE in children disaggregated by sex and age and to examine how the proportion of different DRE types change with age using health insurance claims data from South Korea. The study findings have the potential to influence public health policy and resource allocation for epilepsy management in children, contributing significantly to the understanding and management of this challenging condition.

## 2. Materials and Methods

### 2.1. Study Population and Definitions of Epilepsy and DRE

This study utilized health insurance claims data from the South Korean Health Insurance Review and Assessment Service (HIRA), encompassing 98% of the national population. The dataset included comprehensive information on diagnoses, treatments, and medication prescriptions from all medical institutions [16]. Our analysis focused on pediatric patients under 18 years of age diagnosed with epilepsy between 1 January 2013, and 31 December 2022.

Epilepsy cases were defined as individuals with at least two outpatient, emergency department, or hospitalization visits separated by at least 30 days, with at least one primary, secondary, or additional epilepsy-related diagnosis code from the Korean Standard Classification of Diseases (KCD) (G40 for epilepsy, G41 for status epilepticus, and R56.8 for seizure or convulsion). Additionally, patients must have received anti-seizure medication (ASM) for a minimum of 180 days. This approach is considered one of the standardized methodologies for epilepsy diagnosis [17,18] and enhances diagnostic confidence by cross-referencing the presence of epilepsy-related symptoms with documented medical treatments. ASMs in the study included carbamazepine, oxcarbazepine, clobazam, clonazepam, ethosuximide, phenobarbital, levetiracetam, phenytoin/fosphenytoin, gabapentin, pregabalin, primidone, rufinamide, topiramate, valproate/valproic acid, lamotrigine, vigabatrin, zonisamide, and lacosamide. Notably, the febrile seizures (R56.0) were excluded from this study.

Patients were identified as DRE cases if they received at least one DRE-specific diagnosis code and were prescribed at least three non-gabapentinoid ASMs. This definition aligns with previous research that identified the most specific DRE criteria based on administrative claims data [14]. DRE-related diagnosis codes are detailed in Table S1. Specifically, patients were classified into three types: focal (G40.0x–G40.2x), generalized (G40.3x–G40.7x), and unknown (G40.8x–G40.9x).

### 2.2. Definitions and Calculations of Prevalence and Incidence

Prevalence refers to the total number of cases that meet the established epilepsy criteria within a calendar year. On the other hand, incidence denotes the occurrence of new epilepsy cases within a given year, defined as fulfilling the epilepsy criteria without a prior epilepsy diagnosis or ASM prescription during the preceding observation period. A 2-year lookback period was applied from 2011 to determine epilepsy history. For children under 2 years of age, prevalence and incidence were determined by examining ASM prescriptions and epilepsy-related diagnosis codes from birth until definitive diagnosis.

Prevalent cases of DRE were identified among prevalent epilepsy cases also satisfying the DRE criteria within a given year. Incident cases of DRE were defined as those without prior DRE during the preceding observation period but meeting the criteria for the first time at a specific point in time. A similar 2-year lookback period was applied starting from 2011 to ascertain DRE history. Likewise, prevalent and incident cases among children under 2 years of age were determined by evaluating ASM prescriptions and DRE-related codes from birth until definitive diagnosis.

Annual prevalence and incidence rates for epilepsy or DRE were calculated from 2013 to 2022. The denominator comprised individuals aged 0–17 years obtained from the Korean Statistical Information Service (KOSIS) for each calendar year [19]. The numerators were children under 18 years of age who fulfilled the criteria for prevalent or incident epilepsy or DRE within each calendar year. Crude prevalence and incidence rates were calculated by dividing the number of prevalent or incident cases by the total number of individuals under 18 years of age in a specific year. To correct for annual changes in age distribution, age-standardized incidence rates were calculated by determining age-specific rates in the standard population (the 0–17-year-old population from the 2010 resident register) and weighting the crude incidence rates by these age-specific rates.

Furthermore, annual trends in prevalence and incidence were assessed using APC and AAPC. To reflect direct year-over-year changes, APC was calculated by comparing each year’s rates to the previous year. Meanwhile, AAPC was determined using a log-linear regression model by exponentiating each year’s coefficient. The ratio of annual DRE incidence to annual epilepsy incidence was then determined using age-standardized incidence rates of epilepsy and DRE. The age distribution of prevalent and incident cases by DRE type was also analyzed for each patient, allowing for multiple classifications if both focal and generalized type codes were present.

### 2.3. Statistical Analysis

The 95% confidence intervals (CIs) were determined for prevalence and incidence, and statistical significance was set at *p* < 0.05. SAS Enterprise Guide 9.4.2 ^®^ (SAS Institute Inc., Cary, NC, USA) and R version 4.4.0 (R Foundation for Statistical Computing, Vienna, Austria) were used for all statistical analyses.

## 3. Results

### 3.1. Prevalence and Incidence of Epilepsy and DRE

Between 2013 and 2022, 276,106 pediatric patients aged < 18 years were diagnosed with and treated for epilepsy. The age-standardized prevalence rate of epilepsy increased from 320.1 per 100,000 persons (95% CI, 316.45–323.78) in 2013 to 368.35 per 100,000 persons (95% CI, 363.94–372.81) in 2022 (AAPC = 1.55, *p* < 0.001) (Table 1). This increase in prevalence suggests a steady rise in the number of diagnosed cases over the 10-year observation period. During the same period, 54,057 pediatric patients aged < 18 years were newly diagnosed and treated for epilepsy. The age-standardized incidence rate of epilepsy increased from 66.89 per 100,000 persons (95% CI, 65.25–68.58) in 2013 to 74.06 per 100,000 persons (95% CI, 72.07–76.10) in 2022 (AAPC = 1.53, *p* < 0.01). The incidence data demonstrates an upward trend, indicating an increase in new epilepsy diagnoses annually. Between 2013 and 2022, the standardized prevalence rate of DRE was 3.38 per 1000, and the average standardized incidence rate of DRE was 0.67 per 1000.

Between 2013 and 2022, 21,452 pediatric patients were treated for DRE. The age-standardized prevalence rate of DRE increased from 21.94 per 100,000 persons (95% CI, 21.0–22.92) in 2013 to 31.81 per 100,000 persons (95% CI, 30.52–33.14) in 2022 (AAPC = 4.76%, *p* < 0.001) (Table 2). This indicates a consistent rise in the number of children diagnosed with DRE over the 10-year period. During the same period, 5303 pediatric patients were newly diagnosed with DRE. The age-standardized incidence rate of DRE did not change significantly: 6.73 per 100,000 persons (95% CI, 6.22–7.28) in 2013 and 6.21 per 100,000 persons (95% CI, 5.64–6.80) in 2022 (AAPC = −0.96, *p* = 0.37). Unlike the prevalence rate, the incidence rate remained relatively stable over the study period, showing no significant changes over time. Between 2013 and 2022, the standardized prevalence rate of DRE was 0.26 per 1000 persons, and the average standardized incidence rate of DRE was 0.06 per 1000 persons. The proportion of prevalent cases of DRE among all prevalent epilepsy cases was 8.86% (95% CI, 8.66–9.05), and 9.63% (95% CI, 9.38–9.87) of all incident epilepsy cases were newly diagnosed with DRE.

### 3.2. Age- and Sex-Specific Distribution of Patients with Epilepsy and DRE

**Figure 1:**
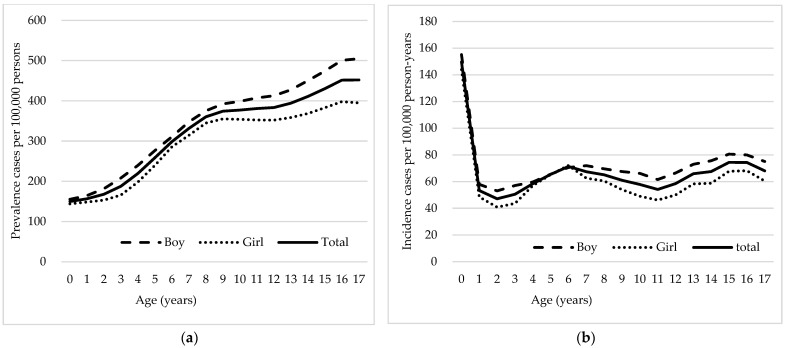
Age-specific distribution of epilepsy prevalence and incidence by sex in Korea from 2013 to 2022. Panel (**a**) shows the age-standardized prevalence of epilepsy per 100,000 persons. Separate lines are used to denote data for boys, girls, and the total population. The graph indicates a higher prevalence among older children and adolescents, particularly boys. Panel (**b**) displays the incidence rates of epilepsy per 100,000 person-years. The graph reveals a peak in the first year of life followed by a decline and stabilization across ages, with boys generally showing higher incidence rates compared with girls.

The age-standardized prevalence of epilepsy was lowest at 149.75 per 100,000 persons in children younger than 1 year (Figure 1a). Prevalence increased steadily with age, albeit at different rates, reaching 452.17 per 100,000 persons at age 17, suggesting a progressive increase in epilepsy prevalence as children grow older. The prevalence of epilepsy was higher among boys than among girls across all ages, and differences between sexes increased with increasing age. The sex difference in prevalence per 100,000 was 11.26 at age < 1 year but increased approximately 10-fold to 109.99 at 17 years. The incidence of epilepsy was the highest in children younger than 1 year, with an age-standardized incidence rate of 149.75 per 100,000 persons, after which the age-standardized incidence rate decreased significantly to 53.31 per 100,000 at 1 year and then increased and decreased with age (minimum 47.11 per 100,000 persons at 2 years; maximum 74.43 per 100,000 persons at 15 years) (Figure 1b). Variations in incidence rates highlight key periods of higher epilepsy onset, notably in early childhood and adolescence. The incidence of epilepsy was also higher among boys across all ages except for ages 5 and 6 years where incidence rates were similar between sexes. The maximum male–female incidence difference was observed at 10 years of age, with a difference of 17.10 per 100,000 persons.

**Figure 2:**
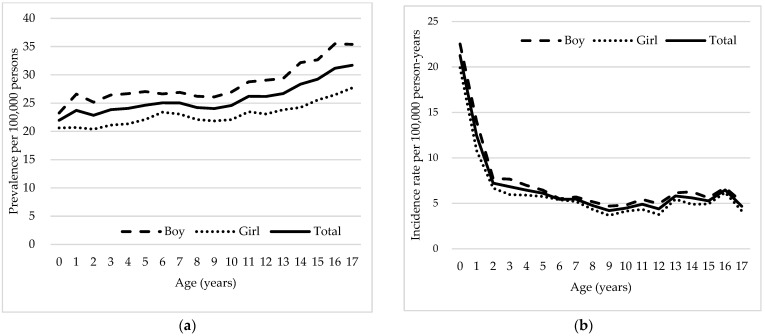
Age and sex-specific prevalence and incidence rates of drug-resistant epilepsy (DRE) in Korea from 2013 to 2022. Panel (**a**) illustrates the age-standardized prevalence of DRE per 100,000 persons, highlighting differences between boys and girls. Panel (**b**) presents the incidence rates of DRE per 100,000 person-years, indicating a peak in early childhood followed by a sharp decline.

The age-standardized prevalence rate of DRE increased from 21.97 per 100,000 persons at age 0 to 31.70 per 100,000 persons at 17 years (Figure 2a). This gradual increase in DRE prevalence with older age suggests an elevated likelihood of DRE diagnosis in older children. Across all ages, a higher prevalence rate of DRE was consistently observed among boys, with a maximum difference of 9.03 per 100,000 persons at 16 years and a minimum difference of 2.65 per 100,000 persons at 0 years. The widening gap in prevalence between boys and girls indicates a potential sex-related disparity persisting throughout childhood and adolescence. Additionally, DRE incidence peaked in early childhood at age 0, with an incidence of 21.15 per 100,000 persons, which steeply declined to 12.31 and 7.10 per 100,000 persons at 1 and 2 years of age, respectively (Figure 2b). The incidence rates continued to decline and were lowest at ages 8–13, after which there was a slight increase in incidence. The incidence of DRE was higher among boys across all ages, but sex differences were not significant (maximum male–female incidence difference of 3.3 per 100,000 persons at age 1). This shows that while boys are slightly more affected, the incidence remains relatively comparable between sexes.

### 3.3. Ratio of Incidence of DRE to Incidence of Epilepsy by Year and Age

**Figure 3:**
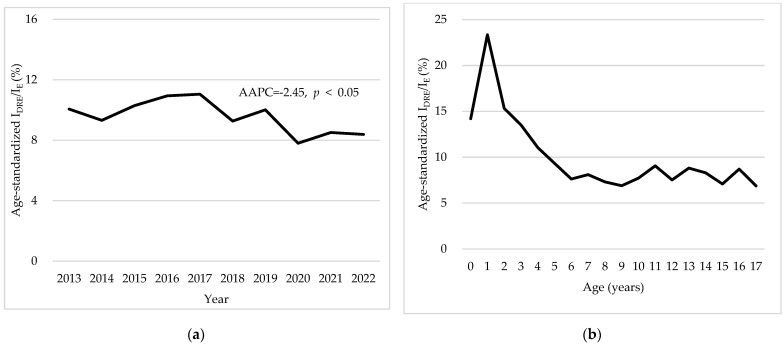
Comparison of the ratio of annual drug-resistant epilepsy (DRE) incidence to the annual epilepsy incidence (I_DRE_/I_E_) per 100,000 persons in Korea from 2013 to 2022. Panel (**a**) presents the annual age-standardized I_DRE_/I_E_ per 100,000 persons, highlighting a decreasing trend over the study period, with an average annual percentage change (AAPC) of −2.45% (*p* < 0.05). Panel (**b**) illustrates the age-specific distribution of I_DRE_/I_E_ per 100,000 persons, showing a peak in the ratio among younger age groups followed by a gradual decrease across older ages. *p*-Values are based on the log-linear regression model. I_DRE_/I_E_: ratio of DRE incidence to epilepsy incidence, DRE: drug-resistant epilepsy, E: epilepsy.

The results regarding the ratio of annual DRE incidence to the annual epilepsy incidence (I_DRE_/I_E_) from 2013 to 2022 are illustrated in Figure 3a. The age-standardized I_DRE_/I_E_ decreased from 10.06% in 2013 to 8.38% in 2022 (AAPC = −2.45, *p* < 0.05), suggesting a gradual decline in the proportion of DRE cases relative to total epilepsy cases over the 10-year period. Figure 3b further presents the age-specific distribution of the I_DRE_/I_E_ ratio. The age group with the highest incidence of DRE relative to the incidence of epilepsy was 1-year-olds (age-standardized I_DRE_/I_E_ = 23.09%), followed by 2- and 0-year-olds. The I_DRE_/I_E_ ratio consistently exceeded 10% until the age of 4 years, indicating a higher proportion of DRE cases in younger children compared with older children. Beyond the age of 4, the ratio stabilized between 7% and 9%, reflecting a lower incidence of DRE relative to total epilepsy with increasing age.

### 3.4. Age-Specific Prevalence and Incidence by DRE Type

**Figure 4:**
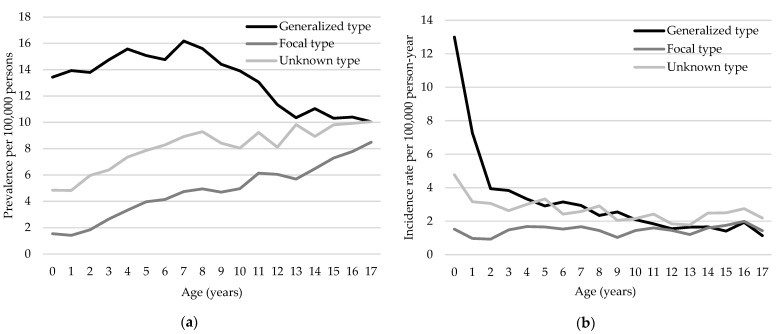
Age-specific prevalence and incidence rates of drug-resistant epilepsy (DRE) types in the pediatric population from 2013 to 2022 in Korea. Panel (**a**) presents age-standardized prevalence rates per 100,000 persons, classified based on DRE types. Panel (**b**) presents the incidence rates per 100,000 person-years, indicating a peak for the generalized type in early childhood, followed by a gradual decline. On the other hand, the incidence rates of the focal and unknown types remain relatively stable. If a patient had both the generalized and focal types, each type was counted individually.

The age-specific distribution of prevalence varied based on DRE type, with the generalized type exhibiting the highest age-standardized prevalence rate. Specifically, this rate peaked at age 7 (16.18 per 100,000 persons), followed by a sharp decline until age 13, remaining relatively stable thereafter at a prevalence of 10.05 per 100,000 persons at age 17 (Figure 4a). The age-standardized prevalence rates for focal and unknown DRE steadily increased from age 0, rising from 1.54 and 4.84 per 100,000 persons, respectively, to 8.49 and 10.05 per 100,000 persons by age 17. The difference in prevalence between the generalized and focal/unknown types steadily decreased after the age of 8 years, with the smallest difference at 17 years. Similarly, the incidence of generalized and unknown type DRE was the highest at age 0 (Figure 4b). However, a gradual decline in age-standardized incidence rate was subsequently observed for the generalized type, whereas the incidence for the focal type remained stable across all ages, ranging from 1–2 per 100,000 person-year. The unknown type showed a steady incidence of approximately 2–3 per 100,000 persons after age 0, indicating a persistent rate of new cases.

## 4. Discussion

This study revealed that, between 2013 and 2020 in South Korea, the prevalence and incidence of pediatric epilepsy gradually increased, the prevalence of DRE increased, and the incidence of DRE did not change. The incidence of DRE gradually decreased compared with the incidence of epilepsy. The prevalence of epilepsy and DRE gradually increased with increasing age. The highest incidence of epilepsy and DRE was observed at age 0, and higher incidence and prevalence rates were observed among boys than among girls across nearly all ages. The prevalence of generalized type DRE was higher at all ages than other types but gradually decreased, while the prevalence of focal and unknown type DRE increased gradually from the first year of life. The incidence of generalized DRE was significantly high at 0 years, while the incidence of focal DRE was relatively constant across all ages.

Between 2013 and 2022, the prevalence and incidence of pediatric epilepsy increased. This result aligns with a Korean population study reporting a similar increase in the prevalence and incidence of epilepsy [20] but contrasts with a study in Taiwan that reported a decrease in the prevalence and incidence of pediatric epilepsy [21]. The increase in the prevalence and incidence of pediatric epilepsy in Korea may be due to improvements in the quality of obstetric and neonatal care, which have increased the survival rate of high-risk newborns [22]. Additionally, the efforts by South Korea to combat negative perceptions of epilepsy, including a name change of epilepsy from “gan-jil” to “noi-jeon-jeung”, celebration of International Epilepsy Day, and preparation of awareness campaigns and educational initiatives, may have reduced the treatment gap and contributed to an increase in disease prevalence and incidence [20,23]. Notably, the prevalence of pediatric epilepsy in South Korea remains comparable to that in developed countries [24] despite the slight increase we observed. The prevalence and incidence rates of epilepsy were higher among boys than among girls, consistent with previous reports from various countries [25]. The prevalence of epilepsy was lowest at 0 years of age and gradually increased with age, with a markedly high incidence at younger than 1 year, followed by a sharp decline and stabilization, consistent with previous studies [25,26,27].

Between 2013 and 2022, the prevalence of pediatric DRE increased, but no significant change in the incidence was observed. Limited research exists on year-to-year changes in the prevalence and incidence of pediatric DREs using national data. The prevalence of DRE was lower in our study than in a population-based study conducted in Italy [28] and lower or similar to that in other population-based studies [5]. A systematic review and meta-analysis of pediatric DRE epidemiology estimated a pooled incidence proportion of 0.15 in children [29], while another study reported a cumulative incidence of 25% and prevalence of 13.7% in general populations and 36.6% in clinic-based cohorts [5]. Similar to the data on epilepsy, our findings revealed that DRE diagnoses were slightly more common among boys than among girls at most ages, but the sex disparity did not worsen with age compared with our observations regarding epilepsy. These results are consistent with previous studies, which show that sex is not a risk factor for DRE [29]. Our study revealed a gradual decline in the ratio of patients with new-onset DRE to those with new-onset epilepsy. The incidence of DRE remained unchanged despite an increase in the incidence of epilepsy. These results may be due to various factors: education and awareness regarding epilepsy through mass media such as YouTube has lowered the stigma, and early detection and intervention have increased the likelihood of a good prognosis [30,31]. In the past, patients with symptoms severe enough to be diagnosed with DRE presumably received treatment at a higher rate than those with less severe symptoms. Therefore, the impact of education and outreach on the incidence of epilepsy is likely greater than that of DRE. Additionally, precision medicine approaches tailored to individual patients through genetic diagnostics may have reduced treatment failure [32]. Despite the development of new ASMs, advances in brain surgery, and various dietary treatments, the prevalence of DRE continues to rise, suggesting that patients who have already developed DRE are increasingly poorly treated and that greater effort is needed to improve patient outcomes [33,34].

The incidence of DRE is markedly higher in the first year of life, accounting for most DRE cases in children and adolescents as epileptic encephalopathies characterized by drug-resistant seizures, developmental delay, and intellectual disability predominantly occur during this time [35]. These include diseases such as early infantile epileptic encephalopathy (Ohtahara syndrome), infantile spasm syndrome (West syndrome), and severe myoclonic epilepsy in infancy (Dravet syndrome), which primarily present with generalized seizures and correlate with the highest proportion of generalized types at age 0 (Figure 4). The incidence of generalized DRE gradually declines and reaches its lowest level in adolescence, similar to that of focal DRE. One study reported that seizure type was not a risk factor for DRE, but in our study, the incidence of generalized DRE was higher than that of focal DRE across most ages, and the prevalence of generalized DRE was the highest across all ages [29]. The prevalence of unknown and focal types of DRE steadily increases with age. This suggests that many patients with unknown or focal epilepsy may experience persistent or worsening symptoms despite the lower initial incidence than generalized types. The prevalence of the generalized type increased slightly from 0 years and then decreased significantly. This change is likely due to a decrease in the incidence of generalized DRE with age and potential improvements or mortality in patients with existing generalized DRE as they age.

This study had several limitations. Because we used insurance claims data, we defined epilepsy and DRE using stricter criteria than clinical definitions (such as only including patients treated with medication), which may have resulted in an underestimation of the prevalence and incidence of epilepsy and DRE. Additionally, misclassification bias is possible with insurance claims data where diagnoses might be coded incorrectly, or patients might be diagnosed with other seizure-related conditions. While ICD codes provide a standardized method and are widely used in epidemiological studies, they may not fully capture the clinical picture of the case. To enhance diagnostic accuracy, we included ASM prescriptions and a number of healthcare visits related to epilepsy in our diagnostic criteria. However, coding errors and differences in clinical practice can still influence case identification (under- or overreporting), emphasizing the need for validation with detailed clinical data. Furthermore, data anonymization precluded the analysis of sociodemographic factors such as regional and social class information. This limitation may have restricted our understanding of the broader social determinants of epilepsy and DRE. In addition, the inability to confirm mortality in the study limited our assessment of prognosis. Future research should consider integrating clinical data with insurance claims data to refine diagnostic accuracy and address misclassification while incorporating socioeconomic factors and longitudinal studies for further elucidation into disease progression and clinical outcomes.

Despite these limitations, this nationwide population-based study determined the prevalence and incidence of pediatric DRE and epilepsy over a 10-year observation period. To date, the epidemiology of epilepsy has been well studied, but the epidemiology of DRE, especially in children, using large-scale data, has been under-researched globally. This is the first epidemiological study of pediatric DRE in Korea using national data, and such studies are rare worldwide.

Although the study provided conservative assessments of DRE incidence and prevalence rates, a significant number of patients have been reported to be diagnosed annually, with prevalence on the rise. As such, clinicians should maintain heightened vigilance when treating infants under 1 year of age due to their increased risk of DRE compared with older children. Increased awareness and comprehensive evaluations are essential for this age group. The consistent occurrence of DRE cases at a steady rate in children over 1 year of age also highlights the need for ongoing preparedness and management strategies. To improve health-seeking behaviors and ensure timely interventions, efforts should be made to promote early diagnosis through public health campaigns. Moreover, further investigation into the factors contributing to DRE development is essential to inform targeted interventions and improve patient outcomes. To that end, policymakers should allocate resources to studies investigating genetic, environmental, and sociodemographic factors affecting DRE incidence.

## 5. Conclusions

This study examined the epidemiology of pediatric epilepsy and DRE in South Korea between 2013 and 2022. The incidence and prevalence of epilepsy exhibited an increasing trend. Although the incidence of DRE has been decreasing, its prevalence continues to increase. Particularly, generalized DRE was the most common type, and DRE incidence was the highest in children aged 1 year or younger. These results underscore the importance of the early detection and treatment of epilepsy and DRE, especially in the first year of life, which is crucial for improving DRE prognosis. Further large-scale studies are needed to evaluate treatment and prognosis in pediatric patients with DRE in Korea.

## Figures and Tables

**Table 1:** Crude and age-standardized prevalence and incidence rates of pediatric epilepsy by year in Korea from 2013 to 2022.

Year	Prevalence					Incidence				
	Case	Crude *	Age-Standardized			Case	Crude *	Age-Standardized		
			A ^+^	AAPC	*p*			B ^+^	AAPC	*p*
2013	29,618	314.03	320.10	1.55	<0.001	6294	66.73	66.89	1.53	<0.01
2014	28,738	312.82	320.05			5670	61.72	61.73		
2015	28,285	315.62	324.30			5693	63.53	63.36		
2016	27,815	318.39	327.41			5540	63.42	63.19		
2017	27,603	325.49	334.41			5469	64.49	65.00		
2018	27,210	332.79	341.37			5340	65.31	66.03		
2019	26,738	337.22	344.40			5262	66.36	67.33		
2020	26,436	342.84	346.53			5208	67.54	68.30		
2021	26,697	356.72	357.17			5290	70.68	71.32		
2022	26,966	370.85	368.35			5323	73.20	74.06		

* Crude prevalence and incidence rate of epilepsy per 100,000 persons. ^+^ Age-standardized annual prevalence (A) and incidence (B) of epilepsy per 100,000 persons. AAPC: average annual percentage change. *p*-Values are based on the log-linear regression model.

**Table 2:** Crude and age-adjusted prevalence and incidence rates of pediatric drug-resistant epilepsy by year in Korea from 2013 to 2022.

Year	Prevalence						Incidence			
	Case	Crude *	Age-Standardized			Case	Crude *	Age-Standardized		
			A ^+^	AAPC	*p*			B ^+^	AAPC	*p*
2013	2057	21.81	21.94	4.76	<0.001	640	6.79	6.73	−0.96	0.37
2014	1938	21.1	21.26			534	5.81	5.75		
2015	1995	22.15	22.32			598	6.67	6.52		
2016	2103	24.07	24.21			619	7.09	6.91		
2017	2147	25.32	25.62			607	7.16	7.18		
2018	2145	26.23	26.49			501	6.13	6.12		
2019	2260	28.5	28.79			527	6.65	6.74		
2020	2201	28.54	28.67			402	5.21	5.33		
2021	2283	30.51	30.5			438	5.85	6.07		
2022	2323	31.95	31.81			437	6.01	6.21		

* Crude prevalence and incidence rate of drug-resistant epilepsy per 100,000 persons. ^+^ Age-standardized annual prevalence (A) and incidence (B) of drug-resistant epilepsy per 100,000 persons. *p*-Values are based on the log-linear regression model.

## Data Availability

We obtained health insurance claims data from the Health Insurance Review and Assessment Service (HIRA) of South Korea for use in our research, and these data are accessible to anyone through approval from the institution. The data provided by HIRA were anonymized and stripped of personally identifiable information before being made available.

## Supplementary Materials

The following supporting information can be downloaded at: https://www.mdpi.com/article/10.3390/neurolint16040066/s1, Table S1: Diagnostic codes associated drug-resistant epilepsy in Korean Standard Classification of Diseases, 8th revision.
