# Supplementary material for: Trends in Prevalence and Incidence of Epilepsy and Drug-Resistant Epilepsy in Children: A Nationwide Population-Based Study in Korea
Source: Neurol Int. 2024 Aug 21;16(4):880–90. doi: 10.3390/neurolint16040066 (PMC11357417; doi:10.3390/neurolint16040066)
Supplement: Supplementary file 1 [file neurolint-16-00066-s001.zip › neurolint-3124315-supplementary.pdf]

Supplement material

Table S1. Diagnostic codes associated drug-resistant epilepsy in Korean Standard Classification of Diseases, 8th revision

KCD code	Category
G40.01	Localization-related (focal)(partial) idiopathic epilepsy and epileptic syndromes with seizures of localized onset with intractable epilepsy
G40.11	Localization-related (focal)(partial) symptomatic epilepsy and epileptic syndromes with simple partial seizures with intractable epilepsy
G40.21	Localization-related (focal)(partial) symptomatic epilepsy and epileptic syndromes with complex partial seizures with intractable epilepsy
G40.31	Generalized idiopathic epilepsy and epileptic syndromes with intractable epilepsy
G40.41	Other generalized epilepsy and epileptic syndromes with intractable epilepsy
G40.51	Special epileptic syndromes with intractable epilepsy
G40.61	Grand mal seizures, unspecified (with or without petit mal), with intractable epilepsy
G40.71	Petit mal, unspecified, without grand mal seizures with intractable epilepsy
G40.81	Other epilepsy with intractable epilepsy
G40.91	Epilepsy, unspecified with intractable epilepsy
